# A training tool for clinicians in segmenting medical images to make 3D models

**DOI:** 10.1097/AS9.0000000000000275

**Published:** 2023-05-23

**Authors:** Soudeh Chegini, Arpan Tahim, Mingjun Liu, Yean Chooi, Eddie Edwards, Matthew Clarkson, Clare Schilling

**Affiliations:** Department of Head and Neck Surgery, University College London Hospital, Wellcome / EPSRC Centre for Interventional and Surgical Sciences (WEISS), University College London; OMFS specialist training, London Deanery; Department of Medical Physics, University College London Hospital; Department of Craniofacial Surgery, Great Ormond Street Hospital; Wellcome / EPSRC Centre for Interventional and Surgical Sciences (WEISS), University College London; Wellcome / EPSRC Centre for Interventional and Surgical Sciences (WEISS), University College London; Head and Neck Academic Centre, University College London

## Abstract

**Introduction:**

3D models produced from medical imaging can be used to plan treatment, design prosthesis, teach and for communication. Despite the clinical benefit, few clinicians have experience of how 3D models are produced.

This is the first study evaluating a training tool to teach clinicians to produce 3D models and reporting the perceived impact on their clinical practice.

**Method:**

Following ethical approval, 10 clinicians completed a bespoke training tool, comprising written and video material alongside online support. Each clinician and 2 technicians (included as control) were sent 3 CT scans and asked to produce 6 fibula 3D models using an open-source software (3Dslicer). The produced models were compared to those produced by the technicians using Hausdorff distance calculation. Thematic analysis was used to study the post-intervention questionnaire.

**Results:**

The mean Hausdorff distance between the final model produced by the clinicians and technicians was 0.65mm SD0.54mm. The first model made by clinicians took a mean time of 1hr 25mins and the final model took 16:04mins (5:00-46:00mins). 100% of learners reported finding the training tool useful and will employ it in future practice.

**Discussion:**

The training tool described in this paper is able to successfully train clinicians to produce fibula models from CT scans. Learners were able to produce comparable models to technicians within an acceptable timeframe. This does not replace technicians. However, the learners perceived this training will allow them to use this technology in more cases, with appropriate case selection and they appreciate the limits of this technology.

## Abbreviations

CT: Computer Tomogram
3D: 3 Dimension
DICOM: Digital Imaging and Communications in Medicine
NHS: National Health Service
FDA: United Stated Food and Drug Administration
CE: Conformité Européenne
ECG: Electrocardiography

## Introduction

Medical imaging is widely used to plan procedures that are customized to the patients individual anatomy. Medical images can be segmented using software to isolate anatomy of interest and produce 3D models. 3D models can be useful in planning complex procedures or in the decision making of suitable procedure for each patient. (1) They are also used to examine the fit of custom implants. They have been shown to improve procedure accuracy and shorten procedure time. They can also be used as a communication tool with patients to allow informed consent and reduce anxiety. (2) Similarly, 3D models have been used to train clinicians. (3) As the public’s awareness of this technology increases, this may become the standard of care expected by patients.

Despite the benefits of 3D models in clinical practice, the use of 3D modelling is not yet standard practice. (2) In most cases the making of 3D models is outsourced to specialist technicians with few clinicians experienced in producing 3D models themselves. It is important for clinicians to understand the limitations of accuracy in how 3D models are produced in order to deploy these appropriately for clinical decision-making. Understanding of a technique demands knowledge of the entirety of the process, (4) and in turn, training clinicians in the use of new technology is known to promote its adoption in the wider profession. (5)

The availability of 3D printers in hospitals makes image segmentation a technology which is ripe for the surgeon to adopt. 3D models can also be appreciated on computers, smart devices and augmented and virtual reality headsets such as Occulus and Hololens.

Since the advent of home computer systems, everyone has used software teaching materials. This is a well studied field which was studied and deliberately applied to develop the tool in this study. (6) Dreyfus-Dreyfus described a five-stage process of skill acquisition, from novice to expert, which has been widely applied in medical education. (7) This was applied in the evaluation of the training tool. Learning how to use a piece of software is a performed ability with results that can be quantitatively evaluated. Therefore, a model used for learning a skill was deemed the most appropriate.

The aim of this study is to evaluate the training tool designed for clinicians in the process of segmentation to produce 3D models. The questions are 1) the impact of the training tool on learning how to make 3D models, 2) the perceived value of the tool in clinical practice. The learners’ models and time taken to produce the models is measured as a surrogate for learning gained using the training tool. The perception and value of the tool is evaluated using a post-intervention questionnaire.

## Material and Methods

Ethical approval was granted by the UCL ethics committee (reference number 19597/001). The study was open to professionally registered dental and medical clinicians aged 25-65, recruited via social media platform.

### Tool Development

For this project a specific training tool was designed to be applied to the open access 3Dslicer software. (8) The tool constitutes structured didactic material, prescriptive exercises and a post-intervention questionnaire. Learners were also supplied with access to download the software and 3 Computerised Tomogram (CT) DICOM files (constitutes 6 fibulas). Digital Imaging and Communications in Medicine (DICOM) is the standard format medical scans can be downloaded and transferred. Each scan contained a left and right fibula within the body of the scan. These were all arterial phase CT angiograms of the lower limbs of 0.75mm^3^ voxel dimension. All scans where acquired from embodi3D, online repository of open access scans for educational and research purposes.

The didactic material was delivered in the form of a written manual, a pre-recorded video and live video-tutorial. All learners complete all three modalities. A blended approach was selected to meet the diverse learning approaches of adult learners and the varied time constraints of busy professionals. This approach was congruent with COVID restrictions.

The didactic material acted as a manual for the segmentation of the first CT scan to produce a 3D fibula model. The production of a fibula bone model was chosen as the example learning case. A bone model was selected due to its distinctive radio-opacity. A single segment model (bone only) is the simplest process for novices to follow whilst learning how to use the software.

The tool consists of a series of steps which instruct the learner to produce a fibula model from CT DICOM. (9) Each step is aligned with a ‘screen capture’ which acts as a complementary stage setting picture. (10) The procedural element provides rigid rules and limits decision making. Discretionary judgment can be difficult and distracting for learners starting from the novice stage as acknowledge in Dreyfus-Dreyfus model of skill acquisition. (7)

Subsequently the learners were given a series of exercises, during which the learners were asked to repeat the steps to produce 5 further fibula models. This was completed in their own time with access to the didactic material and any other notes and sources of their own choosing. Online access to the instructor was available on request. It has been shown that prescriptive exercises have a higher completion rate than ‘on-you-own’ exploration of a software. (11)

The didactic material included declarative commentary alongside the procedural steps. This was relevant on re-reading of the material to complete the exercises. The declarative element provides context within the broader learning of the software abilities and purpose of the task. This holistic view allows the learner to prioritise important aspects and perceive deviation from normal patterns. In this way the didactic material was designed to accommodate for both the novice stage of skill acquisition and the transition to proficiency. (7)

### Data analysis

Two medical physics technicians who regularly produce 3D models were also asked to produce fibula models from the same scans. One technician had 16 years experience and used Robins’ 3D software. The second technician has 8 years experience across clinical and academic image segmentation and used Mimics 3.0 from Materialise in this project. The technicians work at 2 separate large teaching NHS hospitals and produced the models for this study independent of each other. They did not have access to the training tool evaluated in this study. These models were used as the quality control, assuming that they would be of the standard used in regular clinical practice.

Learners submitted the models, as digital files, as they were produced along with the time taken to produce. For analysis, the STL models were imported into Meshlab. (12) The models were then compared using Hausdorff distance analysis. This is an automated analysis tool within the Meshlab software. Hausdorff distance analysis measures how far two subsets of a metric space are from each other. In essence, it utilises the distance between multiple points on the surface of two 3Dmodels. It is commonly used to compare 3Dmodels or objects.

18925 measurement points were used for all Hausdorff distance measurements. Each model pair was measured twice, alternating the base model for the Hausdorff distance measurement. The reported Hausdorff distance measurements in this study are the average between these 2 readings. All analysis was completed by a single un-blinded author. All statistics were calculated using SPSS version 27.

### Post-intervention Questionnaire

An end of course questionnaire was designed to assess the subjective opinion of learners on the value of this tool and its application to clinical practise. This was tested by a series of connected open questions (box 1) which starts with a closed binomial question, then opinion on own practise and then opinion on practise of wider surgical community. This is based on the apprehension, exploration, cooperation, participation interview model. (13) Thematic analysis was applied to analyse the questions 2-5. (14)

## Results

In a one month period 60 persons responded to the study recruitment. Of these 32 consented to participation. Due to study time and staff limitations, 10 learners were invited to undertake the course. All 10 completed the study producing 6 fibula 3D models for inclusion in this analysis.

All participants were between the ages 25-40, 5 male and 5 females. All but 1 participant practised as experienced oral and maxillofacial surgery junior doctors with experience in fibula surgery and 3D printing. None of the participants had any experience with medical image segmentation.

### Variability between technicians

**Figure 1:**
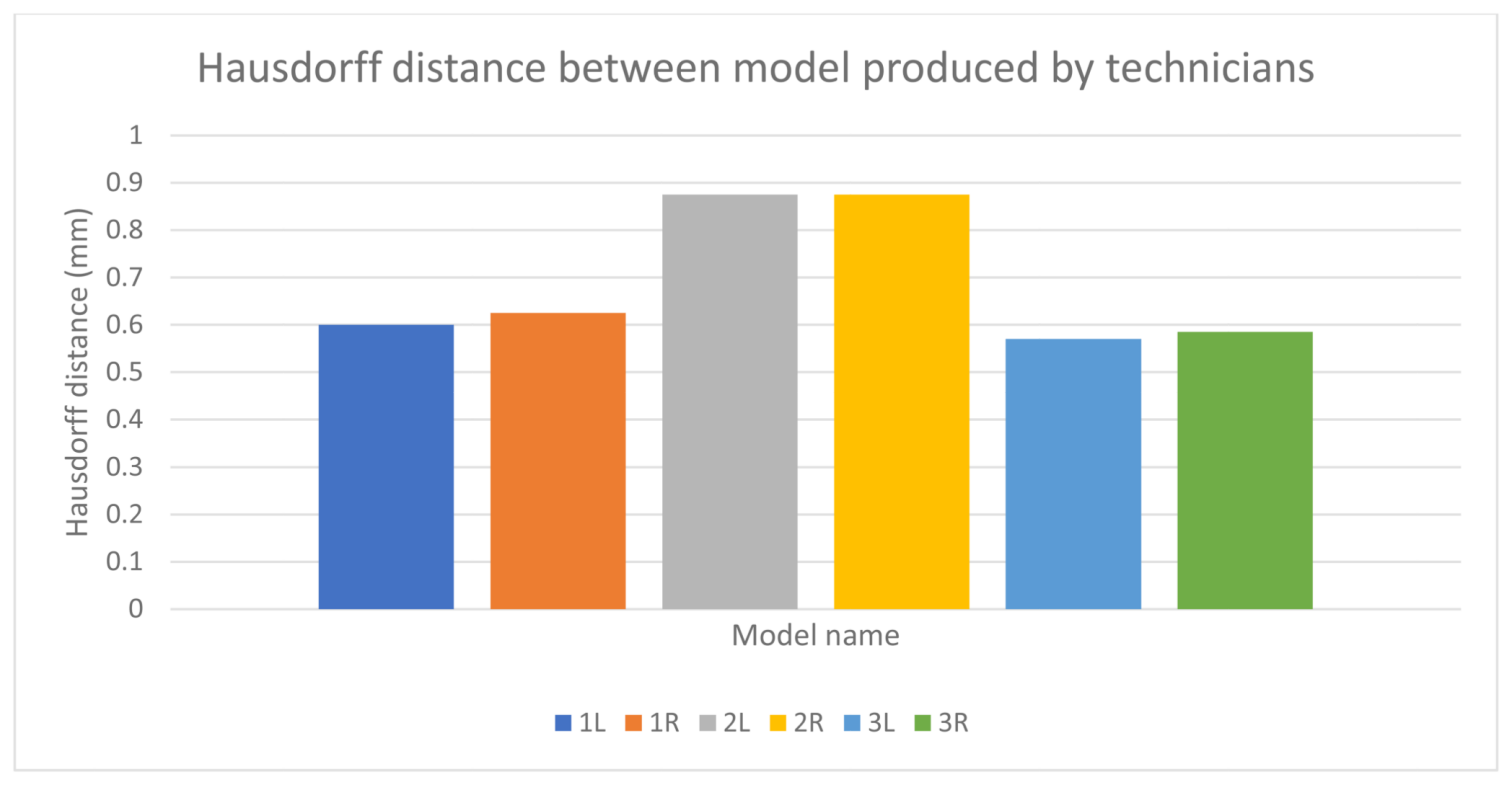
Hausdorff distance between model produced by technician

Figure 1 shows the variation in paired models produced by the 2 experienced technicians. The Hausdorff distance stands at less than 1mm for all models. The second model has the highest Hausdorff distance value suggesting it was the most subjective model to segment.

### Time taken to produce each sequential model

**Figure 2:**
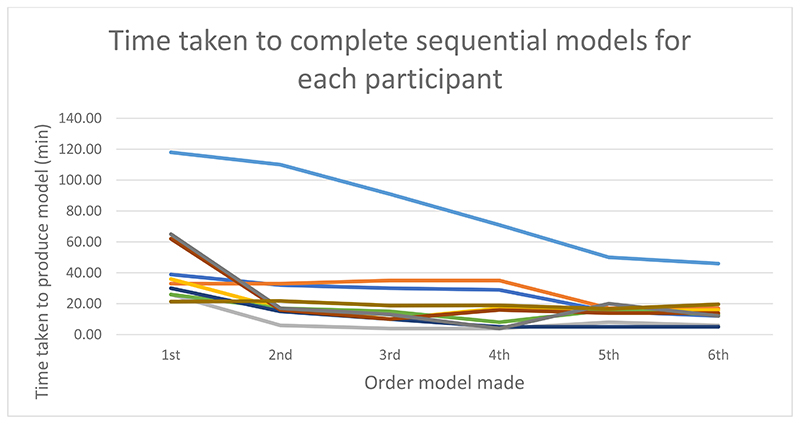
Time taken to produce sequential models. Each colour represents a learner. Proportion is valued as 1 being same time taken to produce 1^st^ model. Values less than 1 represent less time taken to produce a model compared to time taken to produce 1^st^ model

Table 1 and Figure 2 show the time taken to produce models. It can be seen that the mean time taken to produce models decreases over time. A Spearman's correlation was run to determine the relationship between the time taken to produce each model and the order the model was produced. There was a negative monotonic correlation (rs= -.375, n = 60, p =0.003). One learner failed to achieve a sub 30 mins time on 6^th^ iteration. They were offered further practise and were able to achieve this target with their 8^th^ produced model.

### Hausdorff distance between model produced by learners and technician

**Figure 3:**
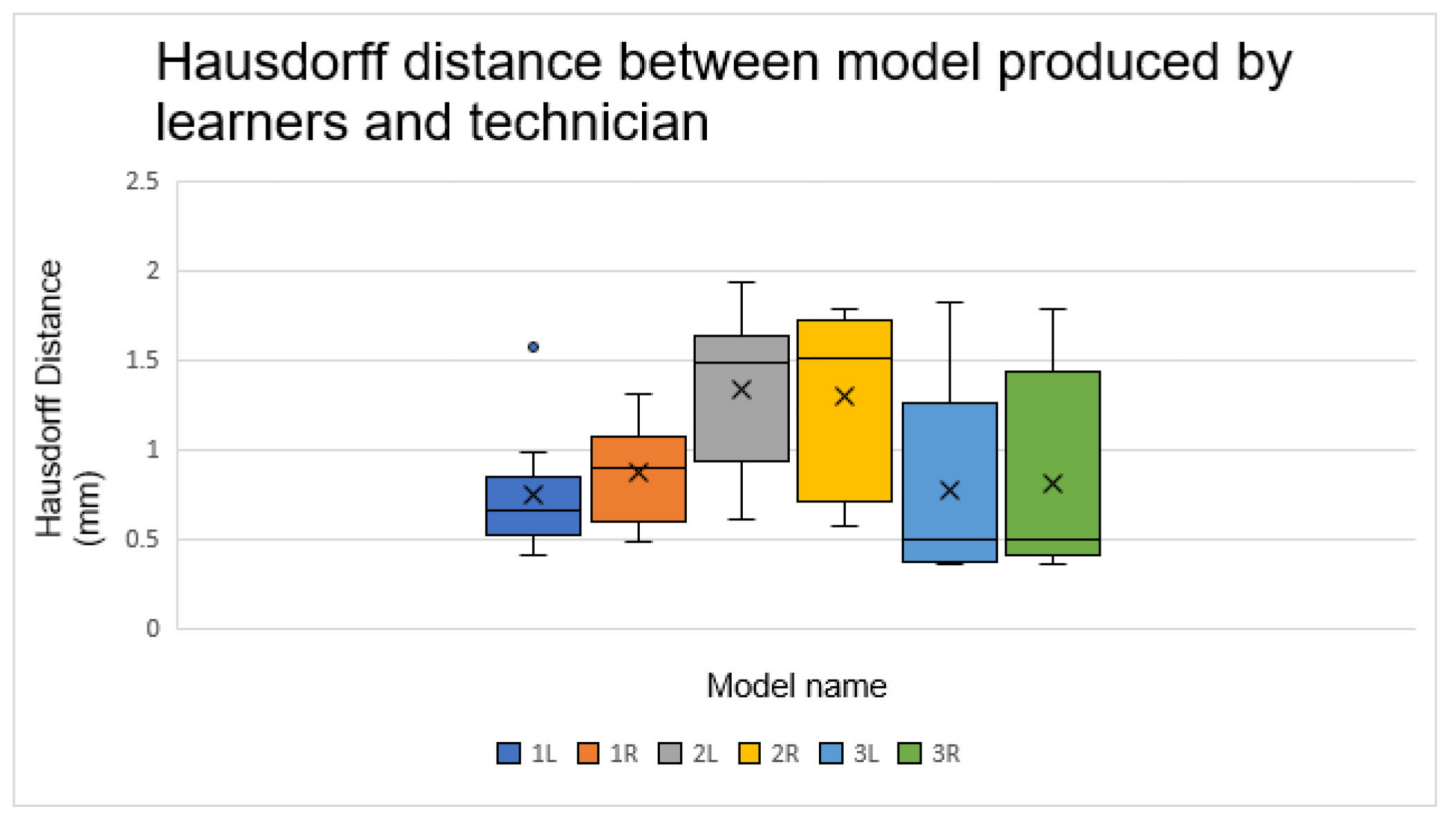
Boxplot showing Hausdorff distance between model produced by learners and technician

Figure 3 shows the mean and range of Hausdorff distance variation by learners. Model 2 shows the greatest model variation. Model 3 which was produced last by learners shows the least model variation.

### Localisation of variation

**Figure 4:**
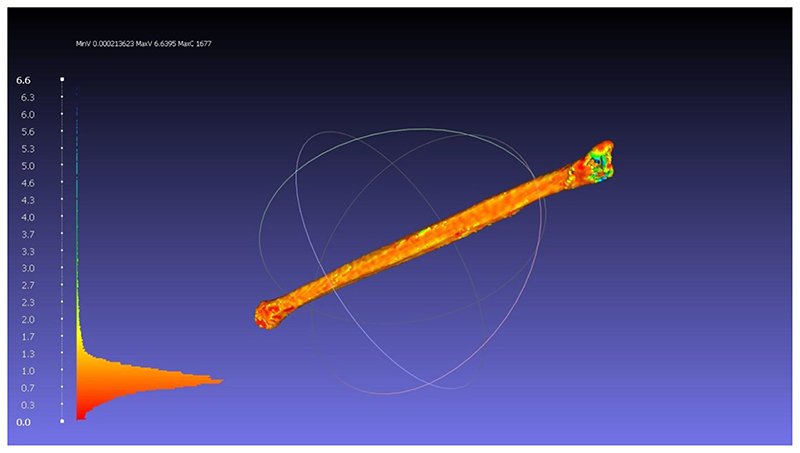
Image taken from meshlab computer software showing the superposition of 2L fibula models. The heatmap shows the areas which are most (green, yellow) and least similar (orange, red) between the models. The histogram on the left shows that red areas have the least difference - nearing 0mm difference. The fibula end and halfway along the shaft where blood vessels are attached to the bone show the most difference in segmentation

Figure 4 is taken from meshlab. The 2L models made by learners and technicians are all superimposed. The heatmap shows the regions of greatest variation between the models (blue/green). The is shown to be at the fibula ends and blood vessel attachment.

### Questionnaire

In response to question 1, all learners reported finding the training tool useful and would use the acquired learning in future clinical practise. Several key themes emerged during the thematic analysis of the qualitative data generated through the questionnaire: Current applications, future impact/benefits and modelling skills. These are shown in table 2.

### Current applications

Learners recognised the benefit of this training both within and beyond their own specialities, listing a variety of procedures. Making their own models was frequently reported as being beneficial to treatment planning.

“It allows us to provide a visual assessment prior to treatment.” (learner 10)

“This software can be used for planning …. three dimensionally by other specialties as well.” (learner 4)

### Future impact/benefits

The enhancement to treatment planning was perceived to be associated with improved clinical outcomes, efficient use of resources and as a communication tool with other professionals and patients. Learners also recognised the cost saving of making their own models compared to outside technical services.

“…. improving patient outcomes and optimising time” (learner 1)

“A software with relatively easy learning curve as this might be time-saving, useful in eliminating the need for third party help, as well as any surprise elements one might come across intraoperatively.” (learner 3)

### Modelling skills

Making models can be time consuming and not suitable for all clinicians. Despite this reality, learners reported many benefits of learning how models are made. This included understanding the limits of this technology, learning terminology and appreciating the variations introduced in the process and in what clinical cases the variation would be unacceptable.

“I have gained some understanding of software option and general terminology” (learner 6)

“Using this software may not be reproducible because the segmentation of the tumour can be subjective.” (learner 5)

## Discussion

The ability to expertly perform a task as a learnt series of steps has been likened to being a skilled technician. Whilst a true expert should be able to tackle novel problems beyond the learnt procedure steps. This is known as adaptive expertise and is a better description of expertise amongst clinicians. Adaptive experts stretch the limits of their experience with flexibility and creativity. In this way they can conquer novel and complex situations. (4) This demands experience of how to perform every step of a task even if those elements are ultimately delegated. During medical training, clinicians are taught how to perform many tasks which are delegated to the multi-disciplinary team. This includes tasks from performing an ECG to setting up surgical equipment in theatre.

The authors are not aware of any publication which evaluates a training tool for clinicians on how to make 3D models from medical imaging. The Radiological Society of North America has held courses on 3D model creation. However, these courses have not been evaluated. Secondly, they are based on the Mimics software which is not open-access and thus not available to all clinicians. (15) Clinicians who wish to employ this technology are dependent on technicians and may not apprehend the limitations of this technology. In turn the technicians do not appreciate the constraints of medical therapy for which they are designing the 3D models.

The authors appreciate that 3Dslicer does not hold FDA or CE approval. (16) However, it is routinely used in research and teaching to make models. It offers comparable ability to its approved counterpart and is an acceptable platform to learn the principles of image segmentation. (8)

The subjective nature of image segmentation is illustrated in the variation in Hausdorff distance between technicians in this study. Whilst small, this variation depends on the quality of medical imaging and the patient’s anatomy. The regions of highest variation are were the structure of interest is close to other structures with the same imaging properties. The heat map of variation (image 1), demonstrates this to be the fibula bone ends. Appreciating the source of these variations and the impact on case selection was highlighted in the responses in the post-intervention feedback.

The tool evaluated in this study was developed based on published understanding of software educational material for novices. Grounding the tool in established knowledge allows the impact of the tool on the target audience to be evaluated rather than testing of the elements within the tool itself. The format of the tool itself is not innovative. However, the development of such a tool for clinicians and measuring the impact on their learning and practice is novel.

The learning of the skill was measured by time taken to produce the models and comparing the models to that produced by technicians. The data shows that learners time taken to produce models statistically reduced as they progressed through the course. Similarly, the models improved. Scan 2 was the first scan segmented by the learners during the ‘prescriptive exercises’. The exercises were performed without supervision. As expected, the analysis of model 2 showed the greatest variation.

The mean difference between the final models made by learners and the technicians was less than that between the technicians themselves. This supports the progression of the learners from novice to proficient as in the Dreyfus-Dreyfus model of skill acquisition. (7) This model is commonly applied to medical education and follows the transition of learners from novice, competent, proficient, expert to mastery. The initial levels require recollection which is represented by learners following the procedural steps within the tool manual. This is following by recognition which is supported by the declarative text within the tool. The latter stages require decision making and awareness. The achievement of these latter stages is supported by the improved final fibula models made by learners.

In the case of the fibula bone, there is variable radiolucency at the bone ends. Depending on the Hounsfield unit applied by the user, sections of the end can be missed and thus excluded from the final model. Another important area is the margins of the bone with other structures. This can be other bones such as the Tibia or blood vessels (in CT angiogram scans). If there is not a clear space between these structures, users have to apply best judgment and select the plane to divide these structures. This illustrates the subjective nature of image segmentation and the introduction of variation in 3D model making. These regions give rise to the variation in the produced models between learners and technicians and the technicians amongst themselves.

Segmenting these regions requires decision making and is subjective. The final models show that the average variation between models made by learners compared to technicians is less than the technicians amongst themselves. This is despite the significantly more experience of the technicians. Using the Dreyfus-Dreyfus model, it can be postulated that the learners have increased awareness and make improved decisions, achieving more accurate models. This can be attributed to their application of their knowledge of anatomy from medical practise.

The post-intervention questionnaire was used to measure the learner perceived impact of the tool on practice. Analysis of the questionnaire showed all learners found the tool useful. Thematic analysis of responses found this to be in a wide range of surgical practice, both in surgical planning and specific procedures. There was also perceived benefit for non-surgical practise including communication with the team and patients, teaching and research.

This study presents a novel training tool, with high learner satisfaction, to teach clinicians to produce 3D models quickly and comparable models to technicians. Learners perceived the application of this skill to improve surgical planning, communication and teaching, their understanding of digital modelling and improve clinical outcomes.

## Figures and Tables

**Table 1:** Time taken to produce model in order of production

Order	Mean (min)	N	Std. Deviation (min)	Std. Error of Mean (min)	Minimum (min)	Maximum (min)	Range (min)
1	45.63	10.00	29.40	9.28	21.37	118.00	96.63
2	28.47	10.00	29.73	9.40	6.00	110.00	104.00
3	23.67	10.00	25.52	8.07	4.00	91.00	87.00
4	20.78	10.00	20.57	6.50	4.00	71.00	67.00
5	17.92	10.00	12.18	3.85	5.00	50.00	45.00
6	16.07	10.00	11.45	3.62	5.00	46.00	41.00
Total	25.42	60.00	23.92	3.08	4.00	118.00	114.00

**Table 2:** Frequency of excerpts representing identified codes in the questionnaire responses

Code	Excerpt Frequency	Theme
Treatment planning	6	Current applications
Saving time	5	Future impact/benefits
Reducing healthcare costs	1	Future impact/benefits
Easy to use software	1	Modelling skills
Eliminating the need for external companies	2	Future impact/benefits
Visualising anatomical structures	4	Future impact/benefits
Improving patient outcomes	3	Future impact/benefits
Software understanding and terminology	1	Modelling skills
MDT communication	1	Future impact/benefits
Teaching	1	Future impact/benefits
Errors in segmentation	2	Modelling skills

## Box 1 Post-intervention Questionnaire items

1. Would you use this software again?
2. What area of practice can you apply this software?
3. And how will this improve your practice? If not, why?
4. What are the benefits of this course for other surgeons?
5. What are the limitations of the applications of this course for other surgeons?

## Mini-abstract

A prospective cohort study evaluating a training tool to teach clinicians to produce 3D models.The mean Hausdorff distance between the final model produced by the learners, compared to models produced by technicians, was 0.65mm SD0.54mm. There was a statistically significant reduction in the time taken to produce models and the final model took 16:04mins (5:00-46:00mins). Thematic analysis of the post-intervention questionnaire was used to record the perceived impact of the tool on clinical practice. 100% of learners reported the training tool as useful in their future practice, in more cases and selecting appropriate case with new learning of the limits of this technology.

## References

[1] Zein NN, Hanouneh IA, Bishop PD, Samaan M, Eghtesad B, Quintini C (2013). Threedimensional print of a liver for preoperative planning in living donor liver transplantation. Liver Transpl.

[2] Diment LE, Thompson MS, Bergmann JHM (2017). Clinical efficacy and effectiveness of 3D printing: a systematic review. BMJ Open.

[3] Waran V, Narayanan V, Karuppiah R, Owen SL, Aziz T (2014). Utility of multimaterial 3D printers in creating models with pathological entities to enhance the training experience of neurosurgeons. J Neurosurg.

[4] Mylopoulos M, Regehr G (2007). Cognitive metaphors of expertise and knowledge: prospects and limitations for medical education. Med Educ.

[5] McCulloch P (2020). The IDEAL framework for ensuring safety and effectiveness of medical devices. BMJ.

[6] Van Der Meij HKJ, Steehouder M (2009). Three Decades of Research and Professional Practice on Printed Software Tutorials for Novices. Technical Communication (Washington).

[7] Dreyfus SE, Dreyfus H (1980). a five-stage model of the mental activities involved in directed skill acquisition.

[8] Fedorov A, Beichel R, Kalpathy-Cramer J, Finet J, Fillion-Robin JC, Pujol S (2012). 3D Slicer as an image computing platform for the Quantitative Imaging Network. Magn Reson Imaging.

[9] DK F (1999). The logical and rhetorial construction of procedural discourse. Technical Communication (Washington).

[10] P J (1984). A handbook of software documentation.

[11] Wiedenbeck SZJ, Nawyn J (2000). An activity-based analysis of hands-on practice methods. Journal of computer assisted learning.

[12] Cignoni P, C M, Corsini M, Dellepiane M, Ganovelli F, Ranzuglia G (2008). MeshLab: an Open-Source Mesh Processing Tool.

[13] Spradley JP (1979). The ethnographic interview.

[14] Braun V, Clarke V (2014). What can “thematic analysis” offer health and wellbeing researchers?. Int J Qual Stud Health Well-being.

[15] Wake N, Alexander AE, Christensen AM, Liacouras PC, Schickel M, Pietila T (2019). Creating patient-specific anatomical models for 3D printing and AR/VR: a supplement for the 2018 Radiological Society of North America (RSNA) hands-on course. 3D Print Med.

[16] Di Prima M, Coburn J, Hwang D, Kelly J, Khairuzzaman A, Ricles L (2016). Additively manufactured medical products - the FDA perspective. 3D Print Med.

